# Transcriptome sequencing and endogenous phytohormone analysis reveal new insights in CPPU controlling fruit development in kiwifruit (*Actinidia chinensis*)

**DOI:** 10.1371/journal.pone.0240355

**Published:** 2020-10-12

**Authors:** Lin Wu, Jianbin Lan, Xiaoxue Xiang, Haiyang Xiang, Zhao Jin, Sadia Khan, Yiqing Liu

**Affiliations:** 1 Institute of Special Plants, Chongqing Key Laboratory of Economic Plant Biotechnology, Chongqing University of Arts and Sciences, Yongchuan, Chongqing, China; 2 Department of Biological Sciences, University of Toronto, Scarborough, Ontario, Canada; 3 College of Horticulture and Gardening, Institute of Horticulture Plants, Yangtze University, Jingzhou, Hubei, China; Institute for Horticultural Plants, China Agricultural University, CHINA

## Abstract

Kiwifruit (*Actinidia chinensis*) is a rich nutritious fruit crop owing to a markedly higher content of vitamin C and minerals. To promote fruit set and to increase the yield of kiwifruit, forchlorfenuron (CPPU) has been widely applied. However, the molecular details regarding CPPU controlling kiwifruit development, especially at the fastest fruit growth stage, remain unknown. In the present study, we measured the effect of CPPU on developmental regulation in red-fleshed kiwifruit (*Actinidia chinensis* ‘Hongyang’). Additionally, a cytological analysis was performed to clarify the precise changes in the cell structure of the CPPU-treated kiwifruits. Moreover, the concentration of endogenous phytohormones, including indoleacetic acid (IAA), zeatin (ZT), gibberellic acid 3 (GA_3_), and abscisic acid (ABA), were measured by Enzyme-linked Immunosorbent Assay (ELISA). Furthermore, RNA-Seq was performed to dissect the complicated molecular mechanisms, with a focus on biosynthesis, metabolism, and signaling compounds, such as endogenous hormones, sugars, and L-ascorbic acid. Our results demonstrated that CPPU treatment not only regulates the size and weight of a single fruit but also improves the quality in ‘Hongyang’ kiwifruit through the accumulation of both soluble sugar and vitamin C. It was also seen that CPPU regulates kiwifruit development by enhancing cell expansion of epidermal cells and parenchyma cells, while, promoting cell division of subepidermal cells. Additionally, CPPU significantly increased the gibberellin and cytokinin biosynthetic pathway and signaling, while repressing auxin and ABA biosynthetic pathway; thus, signaling plays an essential role in CPPU controlling kiwifruit development. Notably, transcriptomic analysis revealed that a total of 2244 genes, including 352 unannotated genes, were differentially expressed in kiwifruits because of CPPU treatment, including 127 transcription factors. These genes are mainly enriched in plant hormone signal transduction, photosynthesis, MAPK signaling pathway, starch and sucrose metabolism, and phenylpropanoid biosynthesis. Overall, our results highlight that CPPU regulation of kiwifruit development is mainly associated with an antagonistic and/or synergistic regulatory role of endogenous phytohormones, and enhancing the energy supply. This provides new insights into the molecular details of CPPU controlling kiwifruit development at the fastest fruit growth stage, which is of agricultural importance for kiwifruit breeding and crop improvement.

## 1. Introduction

After fertilization, the main contributors to fruit development are cell division and cell expansion, which are tightly regulated by genotypes and environmental conditions [1–4]. Recent study has laid the foundation to understand these regulatory factors through genetic analysis and genome-wide gene expression profile detection in various model species, including *Arabidopsis thaliana* and *Solanum lycopersicum* (tomato) [5–7]. Among the regulatory factors, plant hormones play a dominant role in cell proliferation and cell expansion, particularly auxins (IAAs), gibberellic acids (GAs), and cytokinins (CTKs), which participate in controlling the growth of related tissues and determine the fruit size [8, 9]. Interestingly, the fruits of abscisic acid (ABA)-deficient mutants are smaller in size, suggesting that ABA may be involved in regulating fruit development [10]. It has also been reported that a few genes that belong to the biosynthetic pathway and/or signaling of these phytohormones, such as PIN-FORMED (PIN) auxin efflux transport protein 4 (*SlPIN4*), Transport Inhibitor Response 1 (*TIR1*), GA 20-oxidases (*GA20ox*) and 9-cis epoxycarotenoid dioxygenase1 (*NCED1*) [11–14], play a dominant role in controlling fruit development. Additionally, a few master regulators of fruit development were also discovered. For example, the *Fw2*.*2* gene encoding plant-specific and fruit-specific protein, functions in regulating fruit size in tomato [15]. Loss of function of a MADS-box gene, *TOMATO AGAMOUS* (*TAG*)-*LIKE1* (*TAGL1*), resulted in a reduction in pericarp thickness from 25 to 15 layers and an absent inner pericarp [16]. Notably, 4 genes with high expression levels in the early stages of fruit development were also discovered in kiwifruit; these genes encode the homologs of plant metallothionein-like protein, β-subunit of mitochondrial ATP synthase, and two unknown proteins [17].

Kiwifruit (genus *Actinidia*) is native to China, and is a rich nutritious fruit crop because of its high concentration of vitamins, minerals, and amino acids [18–20]. However, the characteristics of fruit shape, size, and flavor are distinct among different *Actinidia* species [21]. Interestingly, some species show impressive fruit performance (such as high vitamin C content, excellent flavor and sweetness), but their fruit weight is below 20 g, e.g., *A*. *arguta*, *A*. *melanandra*, *A*. *macrosperma*, and *A*. *polygama* [21]. To increase the size and weight of kiwifruit, a synthetic cytokinin named N-(2-chloro-4-pyridyl)-N-phenylurea (CPPU) has been widely applied since the 1990s [22]. Notably, the effect of CPPU regulation in fruit development of kiwifruits is strongly related to the concentration of treatment [23]. To date, in order to promote fruit set and increase crop yield, CPPU has also been applied to other horticultural crops, such as grape [24], apple [25] and pear [26]. In addition to increasing fruit weight, application of CPPU may also affect the quality of kiwifruits. Specifically, increased sugar accumulation, reduced acidity, and lower firmness were observed as a result of CPPU treatment in *A*. *deliciosa* ‘Hayward’ [27]. In contrast, a previous study also found that CPPU treatment leads to strongly reduced soluble solid content (SSC) and titratable acids in kiwifruits of *Actinidia arguta* ‘Mitsuko’ [23].

The draft genome of the kiwifruit *A*. *chinensis* ‘Hongyang’ was first reported in 2013 [18]. Afterward, 3 high-quality kiwifruit genomes were described, including 2 varieties of *A*. *chinensis* (‘Red5’ and ‘Hongyang’) [19, 20], and 1 *A*. *eriantha* ‘White’ [28]. Additionally, a few master candidate genes related to regulation of important characteristics (e.g., sweetness, vitamin C, anthocyanin, and volatile compounds) of kiwifruit were also discovered through transcriptomic analysis. For instance, a total of 32,536 genes were found in kiwifruit from seven developmental stages via RNA-seq [29]. Among them, the transcript levels of some sucrose synthesis genes, such as sucrose-6-P synthase (*SP*S), sucrose-6F-phosphate phosphohydrolase (*SPP*), and sucrose synthase (*SuSy*), were gradually increased during fruit development, which might explain the observation that the accumulation of soluble sugars increases in kiwifruits from 1.8% of fresh weight at the S2 stage (70 days after anthesis) to 7.12% of fresh weight (FW) at the S7 stage (140 days after anthesis) [29]. Furthermore, a total of 29,327 alternative splicing (AS) events were identified during kiwifruit development through RNA-seq. A number of those AS events are related to various genes that are involved in ascorbic acid biosynthesis, the biosynthesis and metabolism of carotenoids, the chlorophyll metabolic pathway, and ethylene signaling pathway (e.g., aldonolactonase (*Alase*), nonheme hydroxylase (*CHY*), chlorophyll b reductase (*CBR*), and 1-aminocyclopropane-1-carboxylate oxidase (*ACO*)) [30]. Additionally, the effect of CPPU treatment on the volatile compounds released of ‘Hongyang’ kiwifruit during storage was also revealed. More than 5000 differentially expressed genes (DEGs) were discovered through transcriptomic analysis and were mostly enriched in plant hormone signaling, the ribosome pathway, and RNA transport [22].

These data from the genome and transcriptome of the kiwifruit provide a valuable resource not only for biological discovery but also crop improvement. However, our understanding of the molecular mechanism underlying CPPU controlling kiwifruit development (required at the fastest growth stage) remains quite limited. In the present study, we disassembled this complicated mechanism through (1) analyzing the effects of CPPU on kiwifruit characteristics, including the size and weight of a single fruit and the soluble sugar and vitamin C contents, (2) illustrating the change at the cellular level of the kiwifruit after CPPU treatment, (3) monitoring the concentration of endogenous plant hormones in CPPU-treated kiwifruits, and (4) obtaining a deeper understanding of molecular mechanisms of CPPU treatment in kiwifruit.

## 2. Materials and methods

### 2.1 Plant materials and CPPU treatments

Three-year-old plants of *A*. *chinensis* ‘Hongyang’ were used for the present study and were grown in the orchard of Chongqing University of Arts and Sciences, Chongqing, China. The CPPU treatments were performed as previously described [22]. Briefly, after 20 d (days) of pollination, the fruitlets that had grown on the tree were dipped in 5, 10, 20, and 40 mg L^-1^ of CPPU (Huayueyang Biotechnology Co., Ltd., China) for 10 s. Double-distilled water (ddH_2_O) was used for the control treatment. Kiwifruit samples were collected after 0 d, 20 d, 40 d, 60 d, 80 d, and 120 d of CPPU treatments. For each concentration of CPPU treatment, 10 different kiwifruits at each time point were harvested. All samples were immediately frozen in liquid nitrogen and stored at -80°C.

### 2.2 Testing kiwifruit quality

The phenotypes of kiwifruits at each time point were recorded. Moreover, the weight, the transverse diameter, and longitudinal diameter of single fruit were also measured. In addition, to assess the soluble sugar and vitamin C contents in kiwifruits, the plant soluble sugar content test kit and vitamin C content test kit (Nanjing Jiancheng Bioengineering Institute, Nanjing, Jiangsu, China) were used according to the manufacturer’s protocol, respectively. To test the dry matter content of kiwifruits, the weight of kiwifruits and aluminum cans were measured before drying treatment and were recorded as (A) and (B), respectively. These kiwifruits were placed into the aluminum cans and were desiccated until they reached a constant weight at 70°C; finally, the weights were recorded as (C). Therefore, dry matter content was calculated according to the formula (C—B)/A × 100%. For all results, three technical replicates were performed, and all data represent the mean with standard deviations (n = 10).

### 2.3 Cytological analyses

To unravel how CPPU controls kiwifruit development at the cellular level, after 20 d of 20 mg L^-1^ CPPU treatment, those kiwifruits were sectioned in the transverse plane, and then photographed. These kiwifruits after 20 d of ddH_2_O treatment were used as control. Briefly, samples were taken from the outer pericarp (OP) and related tissues at the middle of the fruits and were immediately immersed in fixative. Then, these samples were cut into 4 μm sections and fixed with FAA [31]. All sections were critically examined and photographed using the Nikon Eclipse E100 microscope and the NIKON DS-U3 digital camera.

### 2.4 Determination of the concentration of endogenous IAA, GA_3_, ZT, and ABA

To evaluate the concentration of auxin (indoleacetic acid, IAA), gibberellin (gibberellic acid 3, GA_3_), cytokinin (zeatin, ZT), and abscisic acid (ABA), enzyme-linked immunosorbent assays were performed. Briefly, fresh flesh (0.2 g) from 0 d, 20 d, 40 d, 60 d, 80 d, 100 d, and 120 d samples of both 20 mg L^-1^ CPPU-treated and ddH_2_O-treated kiwifruits were ground in liquid nitrogen. Then, the powders were mixed with 1.8 mL of phosphate-buffered saline (PBS, pH 7.4) and were centrifuged at 3000 r/min for 20 min. Subsequently, the content of IAA, GA_3_, ZT, as well as ABA were measured using the IAA Elisa Kit, GA_3_ Elisa Kit, ZT Elisa Kit and ABA Elisa Kit (Shanghai Enzyme-linked Biotechnology Co., Ltd. Shanghai, China), respectively [32]. For all results, three technical replicates were performed, and all data represent the mean with standard deviations (n = 3).

### 2.5 RNA extraction and library construction

Total RNA was isolated from kiwifruit samples using EasyPure Plant RNA kit (Huangyueyang Biothechnology Co., Ltd., China). Briefly, total RNA was extracted from 3 kiwifruits after 20 d of 20 mg L^-1^ CPPU treatment and 3 kiwifruits after 20 d of ddH_2_O treatment. RNA quality and amount were measured by an Agilent 2100 Bioanalyzer RNA Nanochip (Agilent, Santa Clara, CA) and NanoDrop 2000 spectrophotometer (Thermo) [32, 33]. Then, the RNA samples were used for the RNA-seq library construction. Briefly, 3 μg of total RNA were used for RNA-seq library construction by the NEBNext Ultra^™^ RNA Library Prep Kit from Illumina (NEC, USA) according to the manufacturer’s protocol, which was performed by ABOROAD (Beijing, China). To obtain high-quality reads, the adaptor and low-quality bases were trimmed. Clean reads were aligned to the kiwifruit reference genome database (http://bioinfo.bti.cornell.edu/cgi-bin/kiwi/home.cgi) [18]. Further, the functions of genes were annotated by Clusters of Orthologous Groups of proteins (COG), Gene Ontology (GO), and Kyoto Encyclopedia of Genes and Genomes database (KEGG) with E-value ≤ 1e^-5^ [34]. The gene expression profiles were measured by the Reads Per Kilobase transcriptome per Million mapped reads (RPKM) methods. Differentially expressed genes (DEGs) were defined on condition of log_2_Ratio ≥ 1 and q (adjusted P value) < 0.05 [35]. Furthermore, metabolic pathway analysis was performed by Mapman software (version 3.5.1R2, http://mapman.gabipd.org) [36]. The transcriptome analysis datasets in this paper are available at NCBI (SRA accession number: PRJNA650143).

### 2.6 Quantitative Real-Time Polymerase Chain Reaction (qRT-PCR) analysis

To test the validation of our RNA-seq databases, the expression levels of 11 randomly selected genes were measured by qRT-PCR using the same RNA samples for the transcriptome analysis. Additionally, expression patterns of 8 candidate genes were monitored in kiwifruits after 0 d, 20 d, 40 d, 60 d, 80 d, 100 d, and 120 d of 20 mg L^-1^ CPPU treatment. The kiwifruits treated with ddH_2_O were used for the control. At each time point, 3 CPPU-treated and 3 ddH_2_O-treated kiwifruit samples were used. Briefly, 0.5 μg of total RNA was using for cDNA synthesis via HiScript^®^ II Q Select RT Supermix (Vazyme, China) [32]. The primers were designed using online software (https://www.ncbi.nlm.nih.gov/tools/primer-blast/index.cgi?LINK_LOC=BlastHome) (S1 Table). The length of all qRT-PCR products was between 150 base pairs (bp) to 200 bp. Before qRT-PCR, the melting peaks and dissociation curves were evaluated. Briefly, qRT-PCR assay were performed in 20 μL of reaction volume on QuantStudio 3 and 5 Real-Time PCR Systems, including 10 μL of 2 × ChamQTM SYBR qPCR Master Mix (Vazyme), 0.6 μL of each primer (10 μM), 2 μL of cDNA, and 6.8 μL of double distilled water (DDW). The reaction condition were set as follows: a hold at 95°C for 5 min, followed by 5 cycles of 95 °C for 10 s, 60 °C for 10 s, and 72 °C for 20 s, and completed with a melting curve analysis program [37]. The relative expression level was calculated based on the 2^-ΔΔCt^ method. *AcActin* (*GI*:*149938963*) was used as an internal reference gene [29, 38].

### 2.7 Data analysis

The significant differences of all data were evaluated by SPSS version 16.0 (SPSS Inc., USA) according to Duncan’s multiple range test (P < 0.05). For all results, three technical replicates were performed, and the results were represented as the mean with standard deviations [33, 37].

## 3. Results

### 3.1 The effect of CPPU treatment on fruit development of *A*. *chinensis* ‘Hongyang’

**Fig 1:**
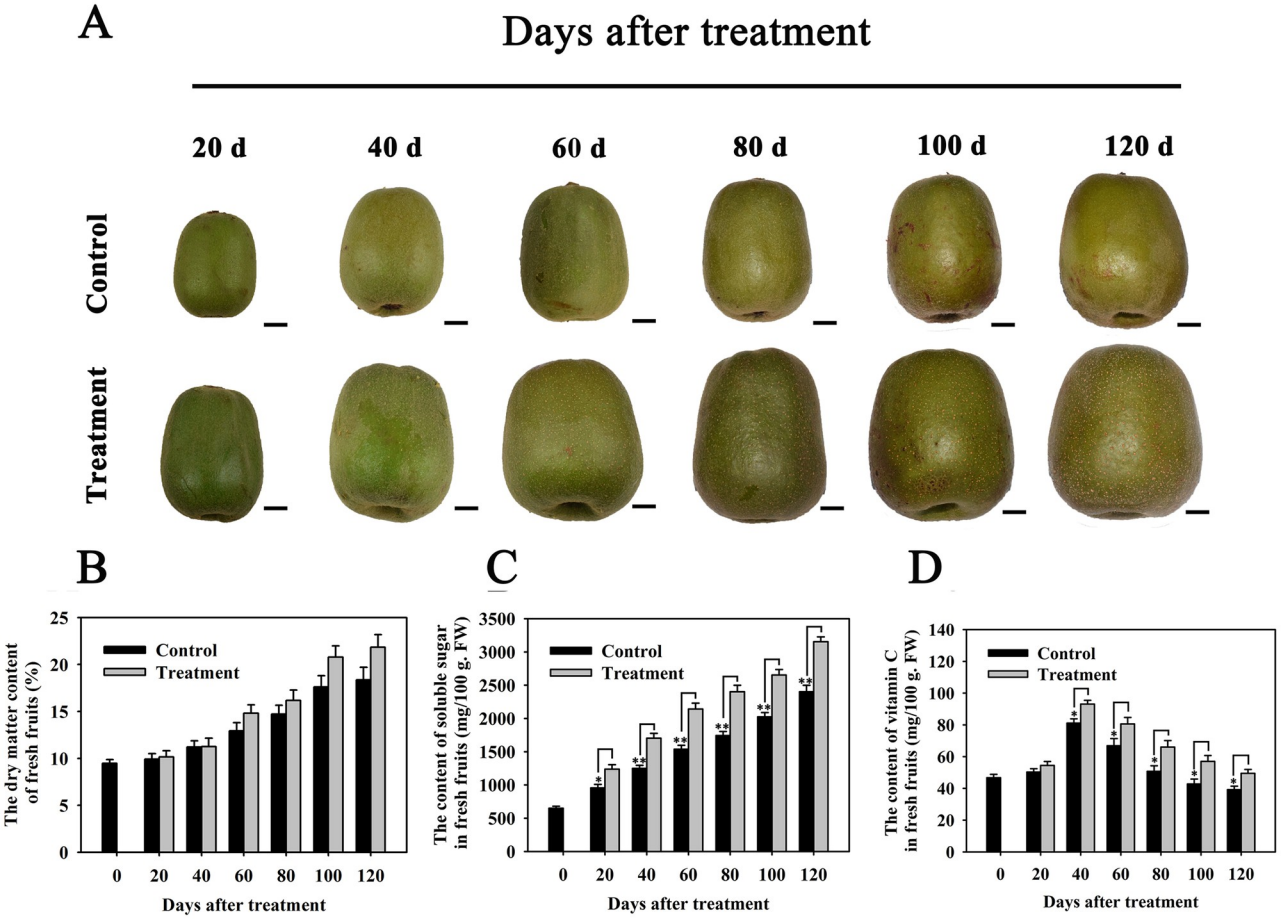
Effect of CPPU on fruit development in *A*. *chinensis* ‘Hongyang’. (A) The phenotype of kiwifruits throughout fruit development. Bar = 1 cm; The contents of dry matter (B), soluble sugar (C), and vitamin C (D) in control fruits and fruits with CPPU treatments during kiwifruit development. In addition, 20 d, 40 d, 60 d, 80 d, 100 d, and 120 d (days) mean the time points after CPPU treatments. For all results, three technical replicates were performed, and all data represent the mean with standard deviations (n = 10). Asterisks indicate a significant difference, * and ** mean p < 0.05 and p < 0.01, respectively.

The role of CPPU in controlling kiwifruit development in different cultivars is tightly related to the application concentration [23, 27]. To determine the appropriate concentrations of CPPU to *A*. *chinensis* ‘Hongyang’, the fresh weight, transverse diameter, and longitudinal diameter of every single fruit (n = 10) was measured every 20 days after CPPU treatments. Compared to the control, we found that 5 mg L^-1^ of CPPU treatment strongly increased both fresh weight and fruit size of single fruit (S1 Fig), and the effect was enhanced with a treatment concentration from 5 to 20 mg L^-1^ of CPPU (S1 Fig). However, no significantly different effects of CPPU controlling kiwifruit development were found between 20 mg L^-1^ and 40 mg L^-1^ of CPPU application. These results suggest that a dose-dependent CPPU regulatory model exists in *A*. *chinensis* ‘Hongyang’, and 20 mg L^-1^ of CPPU was the optimal concentration (Fig 1A). Furthermore, to evaluate the role of the application of CPPU to kiwifruit quality in *A*. *chinensis* ‘Hongyang’, the dry matter content, soluble sugar content as well as vitamin C content in kiwifruits treated with 20 mg L^-1^ CPPU were assessed. Compared to the control, we found that the contents of both soluble sugar and vitamin C in CPPU-treated kiwifruits were significantly increased (Fig 1C and 1D). However, no clear changes in the dry matter content were observed in these kiwifruits after CPPU treatment (Fig 1B). These results indicated that CPPU treatment not only regulated the size and weight of single fruit but also improved the quality of ‘Hongyang’ kiwifruit by accumulation of both soluble sugar and vitamin C.

### 3.2 CPPU regulates fruit size in *A*. *chinensis* ‘Hongyang’ through increasing both cell number and size

**Fig 2:**
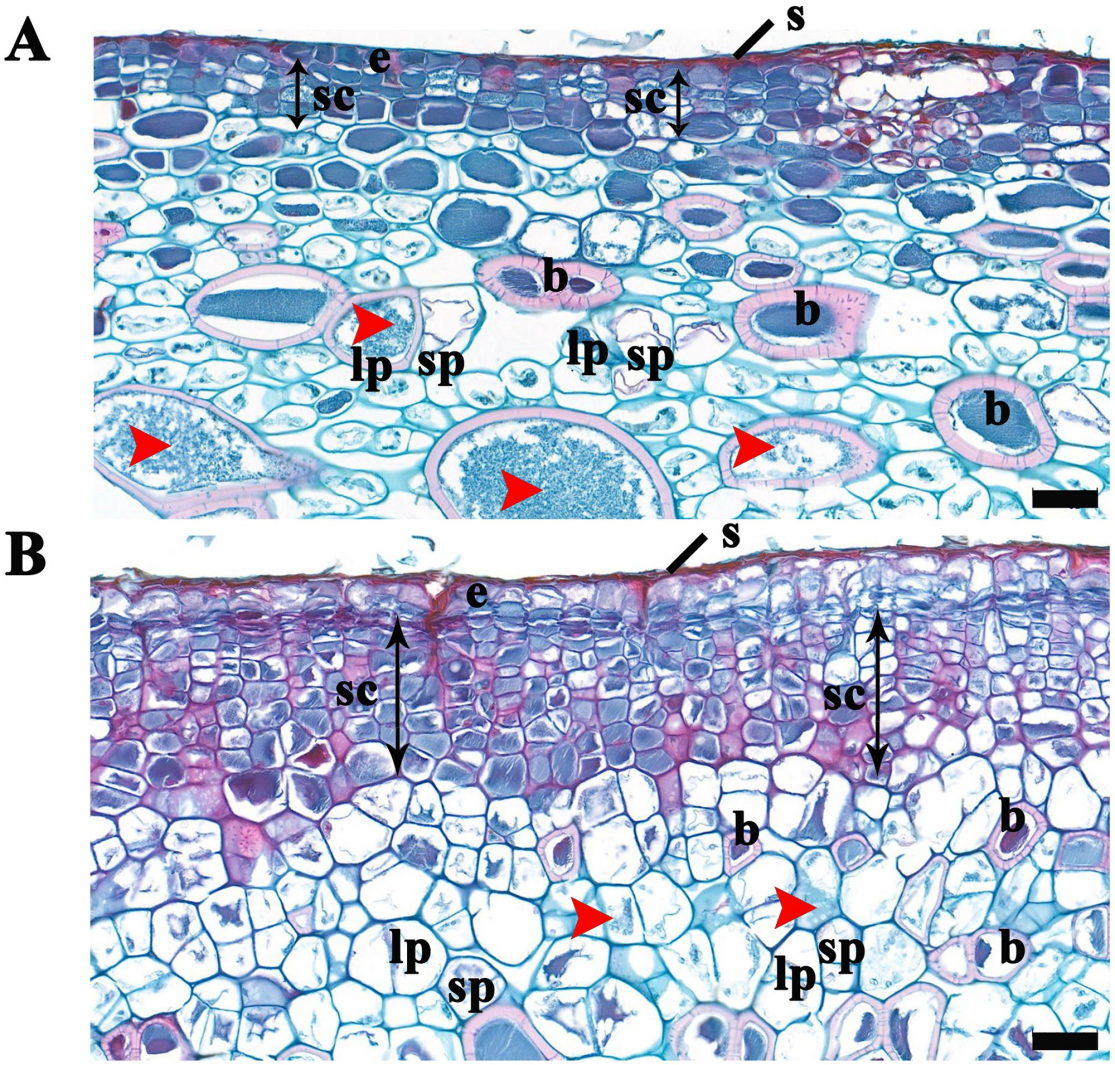
The cell structure in kiwifruits treated with CPPU. Cross section of skin, hypodermis and subhypodermal parenchyma cells in control kiwifruits (A) and in kiwifruits after CPPU treatments (B). Bar = 50 μm, s = dead collapsed skin cell, e = epidermis, sc = subepidermal cell, b = stone cell (brachysclereide), red arrowhead = condensed phenolic material, sp and lp = small and large fresh parenchyma cells.

After fertilization, fruit development and final size rely on both cell division and cell expansion [5–7]. We also found that the weight and size of a single fruit significantly increased in ‘Hongyang’ kiwifruits in response to CPPU treatment (S1 Fig and Fig 1). To understand the precise changes in cell structure in the CPPU-treated kiwifruits, the structure of the skin and associated tissues was observed by cytological analysis. No significant changes to cell structure were found in the outer dead cell layer between control kiwifruits and CPPU-treated kiwifruits (Fig 2). However, beneath the dead skin, the size of epidermal cells was larger in fruits treated with CPPU (Fig 2). Furthermore, the fruits treated with CPPU also had more subepidermal cells and cell layers, which were arranged in a radially seriate manner (Fig 2). Additionally, distal from the skin, the size of both the small and large parenchyma cells were larger in CPPU-treated fruits (Fig 2). In contrast, smaller sizes of stone cells were observed in fruits after CPPU treatment (Fig 2). We also found that the condensed phenolic material significantly decreased in parenchyma cells of the kiwifruits because of CPPU treatment. Our results demonstrated that CPPU regulated kiwifruit size and weight in *A*. *chinensis* ‘Hongyang’ through significantly enhanced cell division and cell expansion.

### 3.3 The influence of CPPU treatment on endogenous phytohormone concentration in kiwifruits

**Fig 3:**
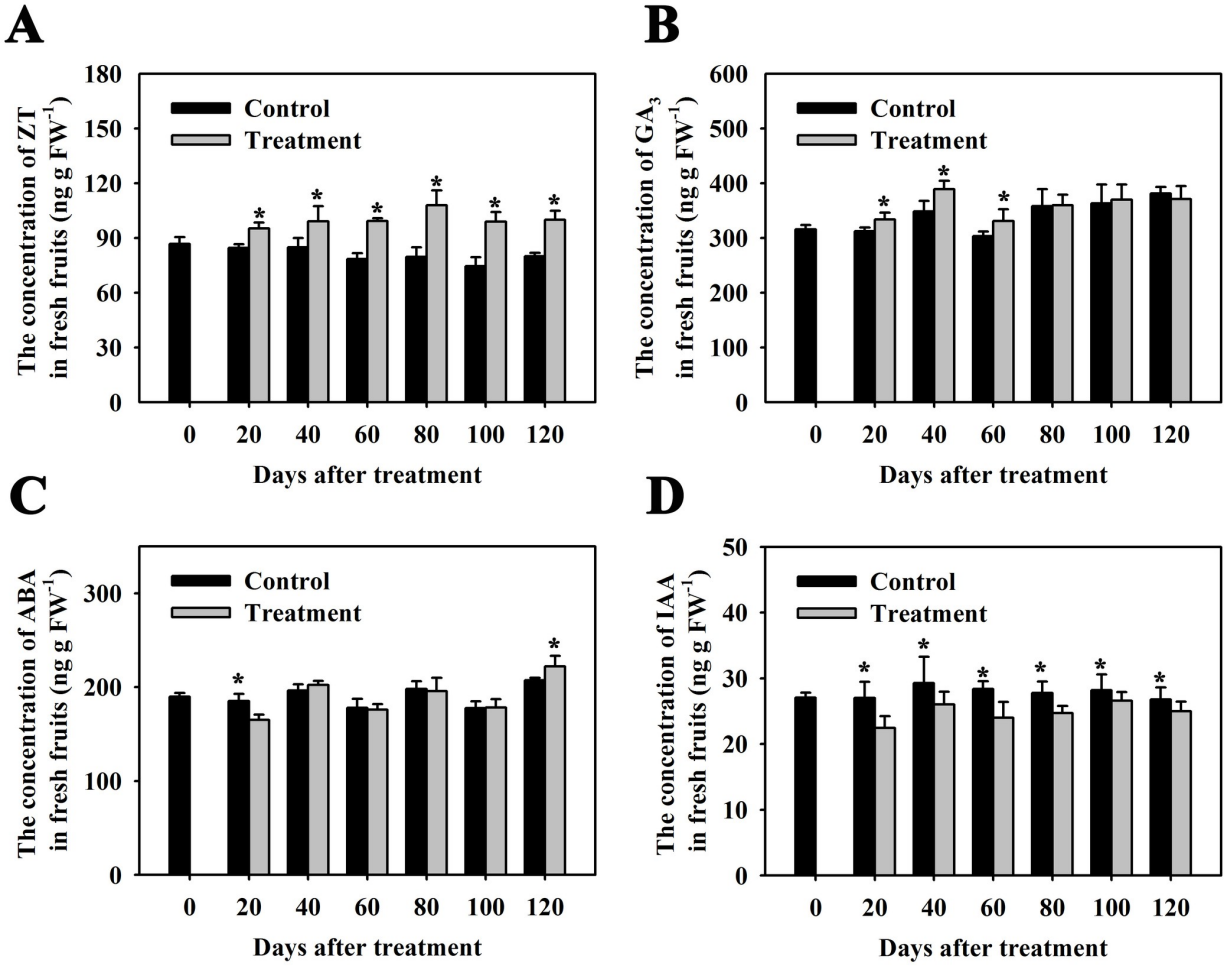
Concentration of ZT, GA3, ABA, and IAA in ‘Hongyang’ kiwifruit in response to CPPU treatment through ELISA. For all results, three technical replicates were performed, and all data represent the mean with standard deviations (n = 3). Asterisks indicate a significant difference, and * means p < 0.05. 0 d, 20 d, 40 d, 60 d, 80 d, 100 d, and 120 d (days) mean the time points after CPPU treatments. FW means fresh weight.

Thus far, a few vital plant hormones that control fruit growth via regulation of cell division and expansion have been illuminated, including auxin, cytokinin, and gibberellin [8, 9]. Indeed, the concentration of these endogenous phytohormones in kiwifruits at seven developmental stages has been monitored by LC-MS [29]. However, the influence of CPPU treatment on those plant hormones is unclear. To figure out whether CPPU-enhanced kiwifruit growth is due to the influence of the content levels of those phytohormones, we measured the concentration of those essential plant hormones by Enzyme-linked Immunosorbent Assay (ELISA), including cytokinin (zeatin, ZT), gibberellin (gibberellic acid 3, GA_3_), abscisic acid (ABA) and auxin (indoleacetic acid, IAA). The concentration of ZT was significantly higher in CPPU-treated kiwifruits than in the control throughout entire fruit developmental phases (Fig 3A). The content of GA_3_ was strongly increased in kiwifruits because of CPPU treatment during the first 60 days after treatment, after which no significant changes were observed (Fig 3B). CPPU treatment decreased ABA concentration levels in kiwifruits after the first 20 days of treatment. After 120 days of treatment with CPPU, the concentration of ABA in kiwifruits was higher than the control (Fig 3C). Interestingly, the concentration of IAA was markedly decreased in CPPU-treated kiwifruits during the entire developmental phase (Fig 3D). According to these results, we determined that CPPU-enhanced kiwifruit growth might influence the concentration of endogenous phytohormones, including significantly increasing ZT and GA_3_ concentrations, while markedly decreasing IAA and ABA contents.

### 3.4 Transcriptional enrichment analysis of CPPU regulation of kiwifruit development

To date, gene expression profiling, long noncoding RNA expression and alternative splicing during kiwifruit development of *A*. *chinensis* have been assessed using transcriptomic sequencing [29, 30]. To better understand the complex mechanisms underlying the role of CPPU in the regulation of kiwifruit development (required at the fastest growth stage), RNA-seq was performed. To reconfirm our RNA-seq data, the expression levels of 11 randomly selected genes were evaluated by qRT-PCR. The same expression patterns were observed between the results of RNA-seq and qRT-PCR, which had a positive correlation (R^2^ = 0.9851) (S2 Fig), indicating that our RNA-seq data were highly reliable.

**Fig 4:**
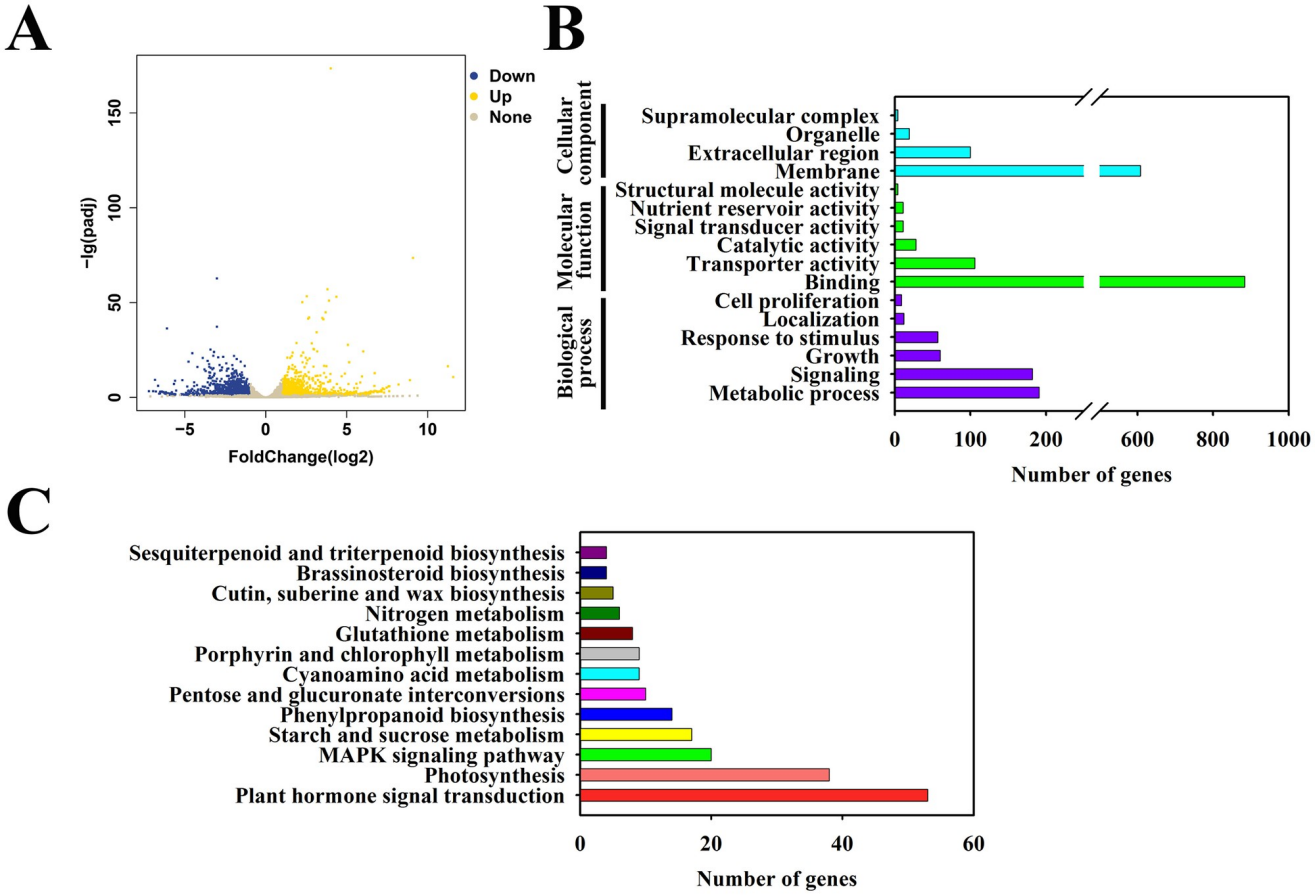
Identification of Differentially Expressed Genes (DEGs) in kiwifruits in response to CPPU treatment. (A) A volcano plot of DEGs. (B) GO enrichment analysis of DEGs. (C) KEGG pathway enrichment analysis of DEGs.

Compared with that of the control, a total of 2244 genes were differentially expressed (DEGs) in the kiwifruits after 20 days of CPPU treatment. Among these genes, 923 and 1321 genes were upregulated and downregulated, respectively (Fig 4A, S2 Table). Interestingly, we also found that 352 (approximately 15.58%) DEGs belonged to unannotated genes, including 185 upregulated genes and 167 downregulated genes (S2 Table).

To further dissect how the upregulation and downregulation of DEGs affect kiwifruit development, we grouped them based on their individual metabolic pathways through gene ontology (GO) enrichment analysis (Fig 4B). After CPPU treatment, these DEGs were divided into three groups: cellular component, molecular function, and biological process. The functions in membrane, binding, and metabolic process, were the most enriched among these three categories (Fig 4B). In addition, biological function pathway enrichment analysis was also constructed via KEGG pathway database. The DEGs in CPPU-treated kiwifruits were mainly enriched in 5 categories, including plant hormone signal transduction, photosynthesis, MAPK signaling pathway, starch and sucrose metabolism, and phenylpropanoid biosynthesis (Fig 4C). These data indicate that CPPU regulation of kiwifruit development occurs mainly through changes to endogenous phytohormone signaling and enhancing the photosynthesis and energy metabolism.

### 3.5 Candidate genes related to phytohormone biosynthesis, metabolism, and signaling involved in CPPU-regulated kiwifruit development

After pollination, phytohormones such as auxin, gibberellins, and cytokinin, play a critical role in controlling fruit development [8, 9]. We also found that the concentration of IAA, GA_3_, ZT and ABA were dramatically changed in the kiwifruits in response to CPPU treatment (Fig 3). To unravel molecular details regarding these genes functioning in the regulating phytohormone biosynthesis, metabolism, and signaling, we screened our RNA-seq datasets. The results exhibited significantly increased concentration of ZT and GA_3_ (Fig 3), additionally both cytokinin and gibberellin biosynthesis and signaling were significantly induced in kiwifruits due to CPPU treatment (Table 1). These results were supported by the increased expression of cytokinin and gibberellin synthesis enzyme genes, such as phosphoribohydrolase ‘Lonely guy’ (*LOG3*) and GA 20-oxidase 1 (*GA20ox1*), and strongly induced expression of cytokinin signaling pathway positive regulators, e.g., *ARR3*, *ARR5*, and *ARR9*. In contrast, dramatically repressed transcripts of the gibberellin signaling negative regulator *DELLA* was also found (Table 1). Furthermore, for ABA biosynthesis and signaling pathway, we found that CPPU treatment significantly repressed *NCED3* expression, which encodes an ABA biosynthetic vital enzyme nine-*cis*-epoxycarotenoid dioxygenase. On the other hand, CPPU treatment markedly induced ABA signaling negative regulator, such as protein phosphatase 2C (*PP2C*). Consistent with a significantly decreased IAA content in CPPU-treated kiwifruits (Fig 3), we also found that the transcripts of 2 *YUC* family genes were decreased, including *YUCCA6* and *YUCCA2-like*, which encode flavin monooxygenases contributing to the tryptophan-dependent auxin biosynthetic pathway (Table 1). Furthermore, the transcripts of 2 auxin influx carrier genes, *LAX1* and *LAX2-like*, were also repressed. Additionally, we discovered that the expression levels of 4 auxin signaling positive regulator genes and 2 auxin-responsive family protein genes (*ARF4*, *ARF5*, *ARF18*, *ARF19-like*, *SAUR32* and *SAUR50*) were also strongly decreased (Table 1). Interestingly, we also found that CPPU treatment significantly decreased the expression of 2 *GH3* genes (*GH3*.*1* and *GH3*.*6*) that encode IAA-amido synthase functioning to conjugate amino acids to auxin (Table 1). Furthermore, expression levels of 3 *PIN* genes (*PIN1-like*, *PIN3*, and *PIN8*), which encode auxin efflux carrier involved in the maintenance auxin gradients, were dramatically repressed (Table 1). The transcripts of 4 *AUX/IAA* genes (e.g., *IAA1*, *IAA26*, *IAA27*, and *IAA27-like*), which act as repressor of auxin-inducible gene expression, were also evidently reduced (Table 1).

**Table 1:** List of DEGs involved in phytohormone biosynthetic pathway and/or signaling in kiwifruits in response to CPPU treatment.

Gene ID	Functional description	Log2-fold change (T VS C)	E value
**Auxin biosynthesis, conjugation, transport, and signaling**			
TRINITY_DN21279_c3_g1	YUCCA6	-3.76	2.12E-03
TRINITY_DN21279_c3_g4	YUCCA2-like	-3.77	7.20E-04
TRINITY_DN20240_c2_g2	GH3.1	-2.89	2.86E-03
TRINITY_DN20571_c1_g2	GH3.6	-3.01	4.48E-06
TRINITY_DN20010_c0_g1	PIN1-like	-2.26	8.69E-08
TRINITY_DN16720_c0_g1	PIN8	-1.32	3.35E-02
TRINITY_DN22190_c0_g1	PIN3	-1.34	4.42E-11
TRINITY_DN19924_c1_g1	LAX1	-3.43	4.03E-04
TRINITY_DN18271_c0_g1	LAX2-like	-1.52	3.28E-02
TRINITY_DN18070_c1_g4	IAA1	-1.35	4.24E-09
TRINITY_DN19433_c0_g2	IAA26-like	-2.02	2.55E-0
TRINITY_DN22693_c1_g5	IAA27	-1.14	1.53E-04
TRINITY_DN18369_c0_g2	IAA27-like	-1.02	4.88E-05
TRINITY_DN22319_c0_g1	ARF4	-1.12	2.64E-03
TRINITY_DN22585_c0_g1	ARF5	-1.23	3.61E-02
TRINITY_DN23253_c1_g3	ARF18	-3.02	1.89E-63
TRINITY_DN16340_c0_g1	ARF19-like	-1.03	4.13E-07
TRINITY_DN19617_c0_g2	SAUR50	-2.63	4.37E-05
TRINITY_DN16997_c1_g1	SAUR32	-2.75	1.00E-04
**Cytokinin biosynthesis and signaling**			
TRINITY_DN14734_c1_g1	LOG1	5.84	1.34e-08
TRINITY_DN22624_c5_g4	ARR3-like	5.45	9.63E-03
TRINITY_DN22624_c5_g6	ARR5-like	3.05	2.42E-02
TRINITY_DN21349_c4_g1	ARR9-like	2.46	4.18E-02
**Gibberellin biosynthesis and signaling**			
TRINITY_DN11848_c0_g1	GA20ox1	9.09	1.80E-78
TRINITY_DN19171_c0_g2	DELLA	-1.01	9.06E-08
**Abscisic acid biosynthesis and signaling**			
TRINITY_DN18340_c1_g2	NCED3	-1.75	2.82E-07
TRINITY_DN12394_c0_g2	PP2C	1.38	1.36E-05
**Ethylene biosynthesis and signaling**			
TRINITY_DN25102_c0_g1	ACO4	6.29	4.32E-03
TRINITY_DN23518_c0_g12	EIN2	-1.00	2.75E-02
TRINITY_DN20745_c0_g1	ETR2	-2.39	8.19E-09
TRINITY_DN22097_c1_g2	ETR2-like	-2.25	3.94E-07
TRINITY_DN22692_c0_g2	RTE1	-1.51	7.30E-04
TRINITY_DN15607_c0_g1	ERF96	1.84	3.02E-03
TRINITY_DN21627_c0_g3	ERF003	4.39	8.33E-08
TRINITY_DN20586_c4_g3	ERF012	2.24	3.77E-05
TRINITY_DN17979_c1_g5	ERF109	2.73	4.05E-03
TRINITY_DN22791_c0_g2	ERF021	2.12	1.73E-04
TRINITY_DN23947_c2_g2	TINY-like	1.80	3.59E-04
TRINITY_DN21196_c0_g1	AP2-like (ANT)	1.33	2.06E-02
TRINITY_DN19535_c1_g1	ERF017	1.05	4.73E-03
TRINITY_DN17937_c1_g2	RAP2-7	-1.73	2.43E-06
TRINITY_DN20735_c3_g2	ERF5	-1.74	2.55E-05
TRINITY_DN16149_c0_g1	ERF23	-1.79	2.54E-02
TRINITY_DN20261_c0_g8	ERF110	-2.14	3.77E-02
TRINITY_DN15282_c0_g1	ERF034	-2.61	5.76E-03
TRINITY_DN17670_c4_g1	ERF12	-3.16	3.01E-13
TRINITY_DN2613_c0_g1	ERF098	-6.33	-1.27E-03

As kiwifruit is a climacteric fruit, ripening is tightly regulated by ethylene [39]. Additionally, it has been reported that CPPU treatment increased ethylene biosynthesis [40], while another study found that CPPU suppressed ethylene production in ‘Xuxiang’ kiwifruits during postharvest ripening [22]. Our data exhibited that CPPU significantly induced ethylene biosynthesis and signaling in ‘Hongyang’ kiwifruits. This result was supported by the increased expression of 1-aminocyclopropane-1-carboxylate oxidase 4 (*ACO4*) encoding a critical ethylene biosynthetic enzyme (Table 1). Consistently, the transcripts of 8 ethylene response genes, *ERF96*, *ERF003*, *ERF012*, *ERF109*, *ERF021*, *TINY-like*, *AP2-like (ANT)*, and *ERF017*, were significantly induced (Table 1). On the other hand, the expression levels of the ethylene signaling negative regulators were dramatically decreased in kiwifruits as a result of CPPU treatment, e.g., *EIN2* as well as *RTE1* (Table 1). Interestingly, we also found that the expression of 2 ethylene receptor genes, *ETR2* and *ETR2-like*, were strongly repressed in CPPU-treated kiwifruits. Notably, a few ethylene response gene expression levels were also significantly repressed in CPPU-treated kiwifruits, including those of *RAP2-7*, *ERF5*, *ERF23*, *ERF110*, *ERF034*, *ERF12*, and *ERF098*. Collectively, these results suggested that CPPU regulated ‘Hongyang’ kiwifruit development mainly through dramatically enhanced biosynthesis and signaling of cytokinin and gibberellin, along with significantly repressed biosynthesis and signaling of abscisic acid and auxin. Additionally, CPPU treatment promoted the ripening of kiwifruits [41], which may be due to markedly enhanced ethylene biosynthesis and signaling.

### 3.6 Induction of genes related to sugar and vitamin C metabolism in kiwifruit resulting from CPPU treatment

Soluble sugars and nonvolatile organic acids are the critical indexes in the flavor of kiwifruits [42], and the high nutritional value of kiwifruits significantly relies on the amount of ascorbic acid (vitamin C) [18]. We also found that both the content of soluble sugar and vitamin C markedly increased in kiwifruits treated with CPPU (Fig 1C and 1D). To understand the molecular details of CPPU regulation in quality of kiwifruits, we screened the key gene functions in the regulation of the biosynthesis and transport of both soluble sugar and vitamin C in our RNA-seq data. We found that the transcripts of 3 positive regulator genes involved in ascorbic acid biosynthesis strongly increased in kiwifruits after CPPU treatment, including 2 L-gulono-1,4-lactone oxidase genes (*AcGULLO3* and *AcGULLO3*) and 1 myoinisitol oxygenase (*AcMIOX1*) (Table 2). Additionally, compared with that of the control, the expression levels of vital genes that function in sucrose biosynthesis and sugar transporter were very clearly increased in kiwifruits in response to CPPU treatment. These essential genes include sucrose synthase 1 (*SUS1*), tonoplast monosaccharide transporter 2 (*TMT2*), hexose carrier protein 6 (*HEX6*), and sugars will eventually be exported transporter 17 (*SWEET17*) (Table 2). Therefore, these results suggested that a major contributor to increased quality in CPPU treated-kiwifruits is the result of significantly changed expression of key genes involved in biosynthesis and transport of both soluble sugar and vitamin C.

**Table 2:** List of DEGs function in sugar and vitamin C biosynthesis and transport in kiwifruits with CPPU treatment.

Gene ID	Functional description	Log2-fold change (T VS C)	E value
TRINITY_DN18346_c1_g2	1.18	GULLO3	6.95E-03
TRINITY_DN17036_c2_g1	2.17	GULLO6	8.25E-03
TRINITY_DN18800_c0_g1	1.41	MIOX1	9.15E-03
TRINITY_DN23465_c0_g1	1.71	SUS1	1.33E-15
TRINITY_DN22345_c2_g1	2.25	TMT2	1.42E-54
TRINITY_DN17138_c1_g12	1.26	HEX6	3.81E-03
TRINITY_DN20191_c7_g1	1.91	SWEET17	4.82E-16

### 3.7 Expression patterns of DEGs associated with plant hormones, sugar, and vitamin C in kiwifruit response to CPPU treatment

**Fig 5:**
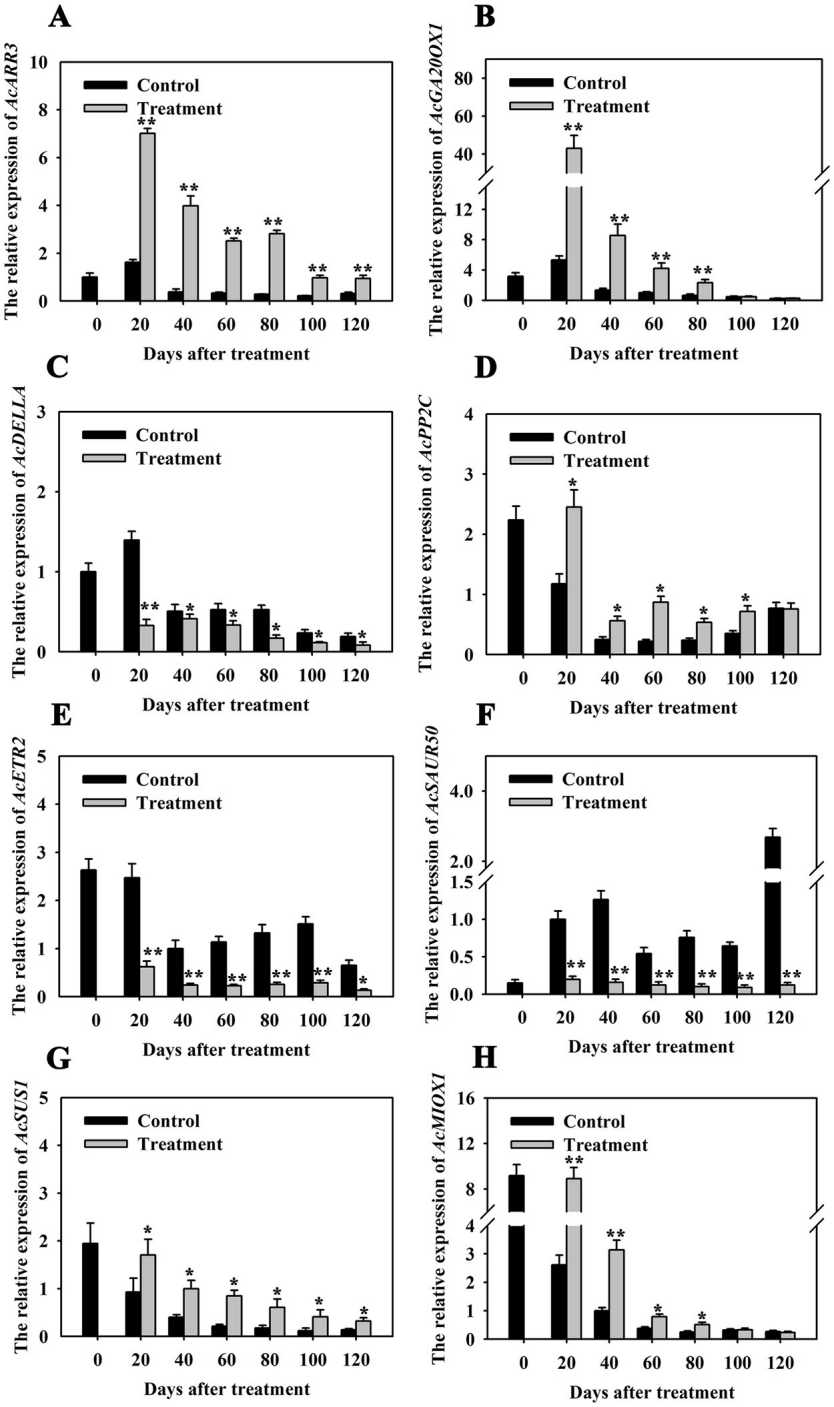
Expression patterns of 8 candidate genes in kiwifruits after CPPU treatment. For all results, three technical replicates were performed, and all data represent the mean with standard deviations (n = 3), and 0 d, 20 d, 40 d, 60 d, 80 d, 100 d, and 120 d (days) mean the time points after CPPU treatments. Asterisks indicate significant difference, * and ** mean p < 0.05 and p < 0.01, respectively.

The concentrations of endogenous phytohormones, sugar, and vitamin C were markedly changed in kiwifruits because of CPPU treatment (Figs 1 and 3). Additionally, these DEGs related to biosynthesis and/or signaling of those secondary metabolites were identified through RNA-seq (Tables 1 and 2). To further monitor the expression patterns of those DEGs in kiwifruits after CPPU treatment, 8 candidate genes related to cytokinin, gibberellin, ABA, ethylene, auxin, sugar, and Vc were selected, including *AcARR3*, *AcGA20ox1*, *AcDELLA*, *AcPP2C*, *AcETR2*, *AcSAUR50*, *AcSUS1*, and *AcMIOX1*, respectively. Compared with the control, and consistent with the content changes of plant hormones, sugar and vitamin C in kiwifruits in response to CPPU treatment; the expression levels of *AcARR3*, *AcGA20ox1*, *AcSUS1*, and A*cMIOX1* were significantly induced (Fig 5). Additionally, the transcripts of *AcDELLA*, *AcPP2C*, and *AcSAUR50* were repressed (Fig 5). Interestingly, after CPPU treatment, the *AcETR2* expression levels were strongly decreased (Fig 5). Overall, these results suggested that CPPU treatment enhanced kiwifruit growth, and increased the contents of both sugar and vitamin C, which were involved in a complex molecular regulatory network.

### 3.8 Activation of transcription factors involved in CPPU regulation of kiwifruit development

**Fig 6:**
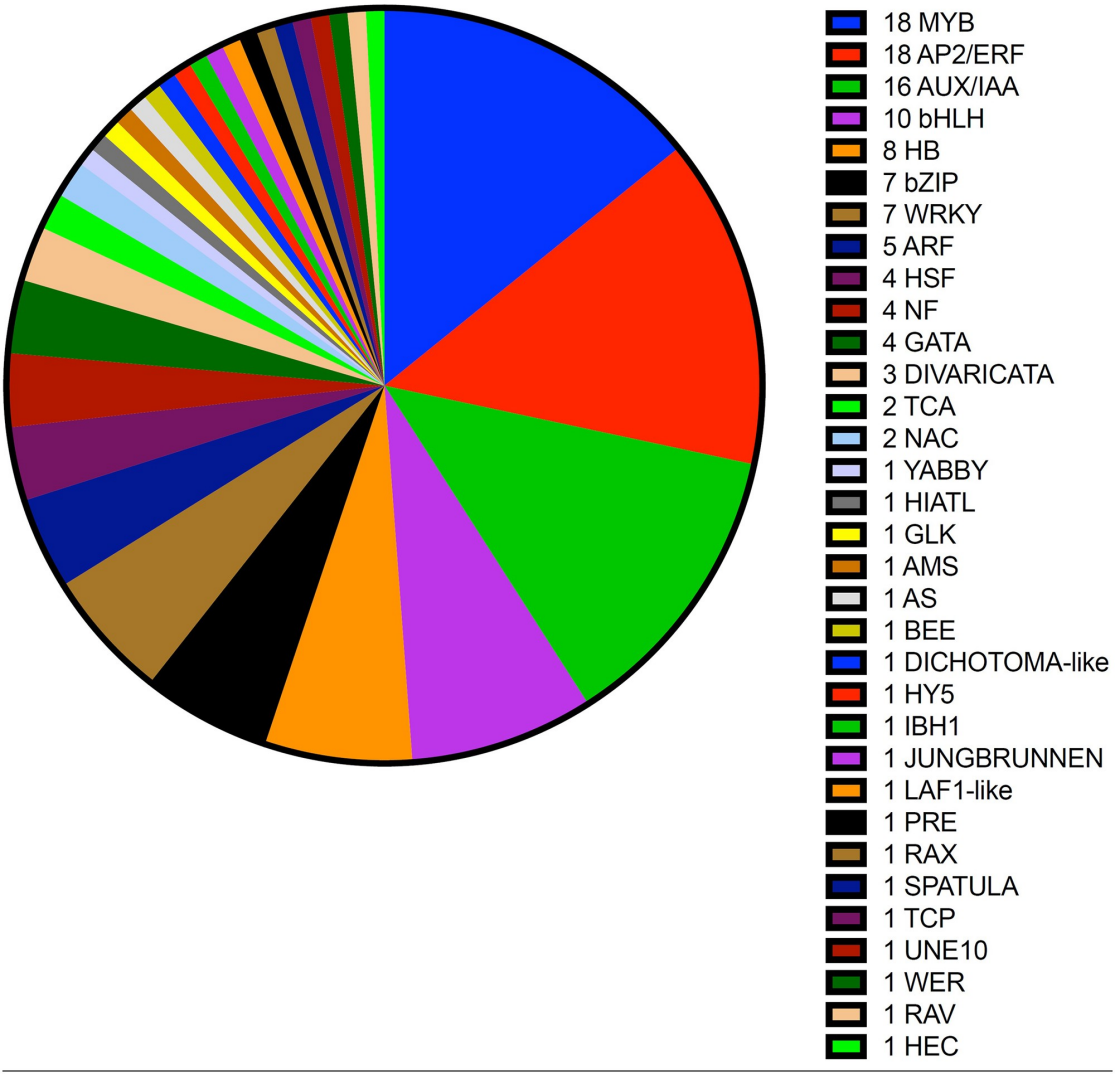
Screening of differentially expressed transcription factors (TFs) in kiwifruits treated with CPPU treatment.

To date, the function of a few TFs in controlling fruit development has been reported in the model plants *Arabidopsis* and tomato. For instance, MADS-box [43], class I KNOTTED1-like homeobox (KNOX) [44], No Apical Meristem/CUP-sharped Cotyledon (NAC) [44], MYB [45], and bHLH [45] are involved in carpel identity and number, fruit patterning, and overall ripening regulation. To understand how CPPU controls kiwifruit development through modification of transcription factor expression, the transcription factors with significant expression changes were examined in our RNA-seq data. A total of 127 transcription factors belonging to 33 different families were differentially expressed in kiwifruits in response to CPPU treatment (Fig 6). Among these TFs, MYB, AP2/ERF, AUX/IAA, bHLH, and HB were the top 5 transcription factor families, including 18, 18, 16, 10, and 8 TFs, respectively. These results indicated that MYB, AP2/ERF, AUX/IAA, bHLH, and HB transcription factors play a critical role in CPPU regulation of kiwifruit development.

## 4. Discussion

To increase fruit set and to improve the yield of horticultural crops including pear, apple, grape and kiwifruit, CPPU has been systematically applied [25, 26]. Interestingly, no significant effect of CPPU on controlling kiwifruit development has been reported in a few *Actinidia* species, such as *A*. *macrosperma* ‘S7’ [21]. In this study, we found both the size and weight of a single kiwifruit of *A*. *chinensis* ‘Hongyang’ strongly increased due to CPPU treatment (S1 Fig). Notably, the effect of CPPU on regulation of kiwifruit development strongly depends on the concentration of CPPU, and an optimal regulatory effect was observed in ‘Hongyang’ kiwifruits treated with 20 mg L^-1^ of CPPU (S1 Fig). This result suggests that a dose dependent regulatory mechanism exists in application of CPPU to control ‘Hongyang’ kiwifruit development. This finding was supported by previous reports that demonstrated that the fruit weight of *A*. *arguta* ‘Mitsuko’ evidently increased as a result of CPPU treatment at concentrations from 1 mg L^-1^ to 10 mg L^-1^, and 5 mg L^-1^ of CPPU was optimal for fruit enlargement [23]. Additionally, the diameter and mass of berries significantly increased in *V*. *vinifera* ‘Himrod’ under different concentrations of CPPU treatment, and fruits with an application of 15 mg L^-1^ of CPPU were consistently greater in size and mass than those with a treatment of 5 mg L^-1^, while no significant difference was observed between fruits with a 10 mg L^-1^ and 15 mg L^-1^ of CPPU treatment [24]. We also observed an increased size of epidermal cells, as well as parenchyma cells, and a greater number and layers of subepidermal cells in kiwifruits because of CPPU treatment (Fig 2). Compared with that of the controls, this outcome may contribute to another understanding of why the weight and size of single CPPU-treated kiwifruit were significantly increased.

Additionally, deep transcriptomic sequencing was performed for dissecting the genetic and molecular mechanisms underlying CPPU control of kiwifruit development at the fastest growth stage. A total of 2244 DEGs were identified for kiwifruits as a result of CPPU treatment. Interestingly, 352 (approximately 15.58%) DEGs are unannotated (Fig 4). The reason for this finding might be that some genes specifically were induced in kiwifruits after CPPU treatment. Similarly, in CPPU-treated ‘Xuxiang’ kiwifruits, only 60.12–66.68% of genes could be mapped [22]. Furthermore, it might be that the reference genome of ‘Hongyang’ kiwifruit is incomplete. This point was also supported by a previous report that 2980 novel protein-coding genes were unannotated in nine fruit RNA-seq libraries of *A*. *chinensis* ‘Hongyang’ [30]. Therefore, these new unannotated transcripts in our study provide a stepping stone to improve the original genomic draft of ‘Hongyang’ kiwifruit.

Furthermore, we observed that the soluble sugar and vitamin C contents were strongly increased in CPPU-treated ‘Hongyang’ kiwifruits, suggesting that CPPU treatment improved not only the yield but also the quality of ‘Hongyang’ kiwifruits (Fig 1 and S1 Fig). Similarly, it has also been reported that the application of CPPU can lead to a lower total titratable acidity and the higher content of soluble solids and soluble sugars in *A*. *deliciosa* ‘Hayward’ [27]. Consistently, the expression levels of a few genes that function in biosynthesis and transport of sugar and/or vitamin C were greatly enhanced in kiwifruits in response to CPPU treatment, including *GULLO3*, *GULLO6*, *MIOX1*, *SUS1*, *TMT2*, *HEX6*, and *SWEET17* (Table 2). In addition, our qRT-PCR results also showed that *AcSUS1* expression was significantly higher in CPPU-treated kiwifruits than in the control during the entire fruit developmental phase (Fig 5). The transcripts of *AcMIOX1* were also markedly induced in kiwifruits from 20 days to 80 days after CPPU treatment (Fig 5). These results were in agreement with previous findings that the *GULLO* gene encoding L-gulono-1,4-lactone oxidase plays a key role in oxidizing of L-gulono-1,4-lactone (*L-GulL*) to AsA [46]. Furthermore, transcripts of the *SUS* gene, which encoded a protein with sucrose synthase activity, gradually increased during kiwifruit development [29].

After fertilization, fruit development consists of fruit set, fruit growth, maturation, and ripening [4]. During the fruit growth phase, both initial active cell division and eventual later cell expansion synergistically determines final maximum size, which is strictly regulated by phytohormones in the fruits [1–4]. Among them, auxins, GA, ABA, and CTKs are seen to be the most critical hormones during this developmental phase [1–4]. We also found that the concentration of gibberellin and cytokinin were strongly increased in CPPU-treated kiwifruits (Fig 3). By contrast, ABA and auxin contents were clearly decreased (Fig 3). These data suggested that CPPU treatment could regulate kiwifruit growth through influencing the biosynthesis of gibberellin, cytokinin, ABA and auxin. This point was also supported by our transcriptomic data. We found significantly increased expression levels of genes encoding GA and CK biosynthetic key enzymes, such as *GA 20-oxidase 1* (*GA20ox1*) and cytokinin phosphoribohydrolase ‘Lonely guy’ (*LOG3*) (Table 1). These results were similar to previous reports that upregulation of *GA20oxs* contributed to active GAs accumulation, which is required for fruit development in tomato [47, 48]. The expression of *SlLOG2*, a CK biosynthetic gene, significantly increased after anthesis [49]. We also determined repressed gene transcripts encoding auxin and ABA biosynthetic critical enzymes, containing flavin monooxygenase (*YUCC2* and *YUCCA2-like*) and nine-cis-epoxycarotenoid dioxygenase (*NCED3*) (Table 1). Similarly, it has also been reported that the contents of ABA and auxin were at higher levels in the nascent ‘Hongyang’ kiwifruits and then experienced a low level during the fastest growth stage from 30 to 70 days after anthesis (DAA) [29]. This finding indicates that low concentration of ABA and auxin during kiwifruit growth phase is required for cell division and cell expansion. It was also supported by *NCED* gene expression, which was significantly reduced in tomato during fruit growth stage [50]. Strikingly, the accumulation of auxin and activation of auxin signaling system were also observed in a few developmental fruits. For example, two ARF gene expressions were induced in the tomato pericarp after 5 days of pollination [51], suggesting that a different regulatory mechanism of auxin in controlling fruit development might exist in kiwifruit and tomato.

Interestingly, in our case, we also discovered dramatically activated signaling of cytokinin and gibberellin, while reporting repressed signaling of ABA and auxin in kiwifruits in response to CPPU treatment. For example, strongly increased expression levels of *ARR3*, *ARR5*, and *ARR9*, and *PP2C* were found in CPPU-treated kiwifruits (Table 1). In contrast, CPPU treatment significantly repressed the expression of *DELLA*, *ARF4*, *ARF5*, *ARF18*, *ARF19-like*, *SAUR32* and *SAUR50*. These findings agreed with previous reports that a reduction in *DELLA* activity is thought to promote the parthenocarpic fruit growth [48]. Likewise, type-A response regulator genes have also been found to be highly expressed in tomato ovaries from preanthesis to early postanthesis stages [49].

Interestingly, in response to CPPU treatment, ethylene biosynthesis and signaling were activated. This outcome was supported by the finding of strongly increased expression of *ACS4*, *RAP2-7*, *ERF5*, *ERF23*, *ERF110*, *ERF034*, *ERF12*, and *ERF098*, with dramatically repressed expression of *EIN2* and *RTE1* (Table 1). This information may supply molecular evidence that CPPU significantly improves fruit softening during maturation and postharvest storage [52].

We also found transcription levels of 127 transcription factors belonging to 33 different families in kiwifruits strongly changed after CPPU treatment, including MYB, AP2/ERF, AUX/IAA, bHLH, and HB (Fig 6). In fact, earlier studies at the molecular level had established that AUX/IAA protein plays a role in governing the fate of fruit development. For example, IAA9 and ARF8 may form a transcriptional repressor complex, which is implicated in parthenocarpic fruit formation in tomato and *Arabidopsis* [53]. Biochemical and molecular data have demonstrated that MYB and bHLH transcription factors play an important role in regulation of the flavonoid biosynthesis in several species, including tomato, grape, apple, pear, and strawberry [54]. Moreover, HD-Zip homeobox protein and AP2/ERF proteins, such as LeHB-1, SlAP2a and SlERF6 have been shown to function in regulation of fruit development, especially in the ripening stage, [55–57]. Here, our data may supply new proof of the involvement of MYB, AP2/ERF, bHLH and HB transcription factors in regulating fruit development during cell division and cell expansion phases.

## 5. Conclusions

In the present study, our results demonstrated that application of 20 mg L^-1^ of CPPU significantly improved fruit development in ‘Hongyang’ kiwifruit. These improvements include an increased size and weight of a single fruit, and the accumulation of soluble sugar and vitamin C. Additionally, CPPU contributed to the increased kiwifruit size by enhancing cell expansion of epidermal cells and parenchyma cells, in addition to promoting cell division of subepidermal cells. Furthermore, CPPU controls kiwifruit development mainly through synergistic and/or an antagonistic regulatory role with the endogenous phytohormones, including auxin, cytokinin, gibberellin, abscisic acid, and ethylene. Finally, MYB, AP2/ERF, AUX/IAA, bHLH, and HB transcription factors may be involved in CPPU regulation of kiwifruit development.

## Supporting information

S1 Fig Effects of CPPU on the single fruit weight and fruit size of ‘Hongyang’ kiwifruit. (TIF)

S2 Fig Expression levels of 11 DEGs genes in kiwifruit in response to CPPU treatment. (TIF)

S1 Table DEGs genes in CPPU-treated kiwifruits. (XLSX)

S2 Table Primer list. (DOCX)
