# Supplementary figures and images for: Transcriptome sequencing and endogenous phytohormone analysis reveal new insights in CPPU controlling fruit development in kiwifruit (Actinidia chinensis)
Source: PLoS One. 2020 Oct 12;15(10):e0240355. doi: 10.1371/journal.pone.0240355 (PMC7549808; doi:10.1371/journal.pone.0240355)

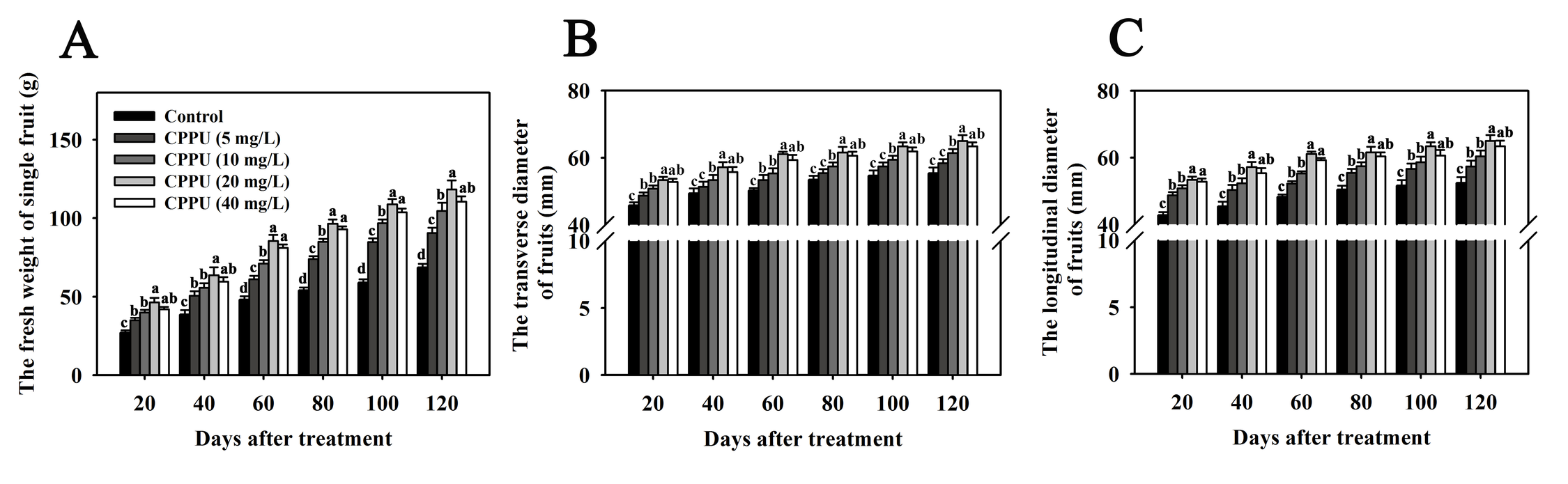

Supplement: S1 Fig — (TIF) [file pone.0240355.s001.tif]

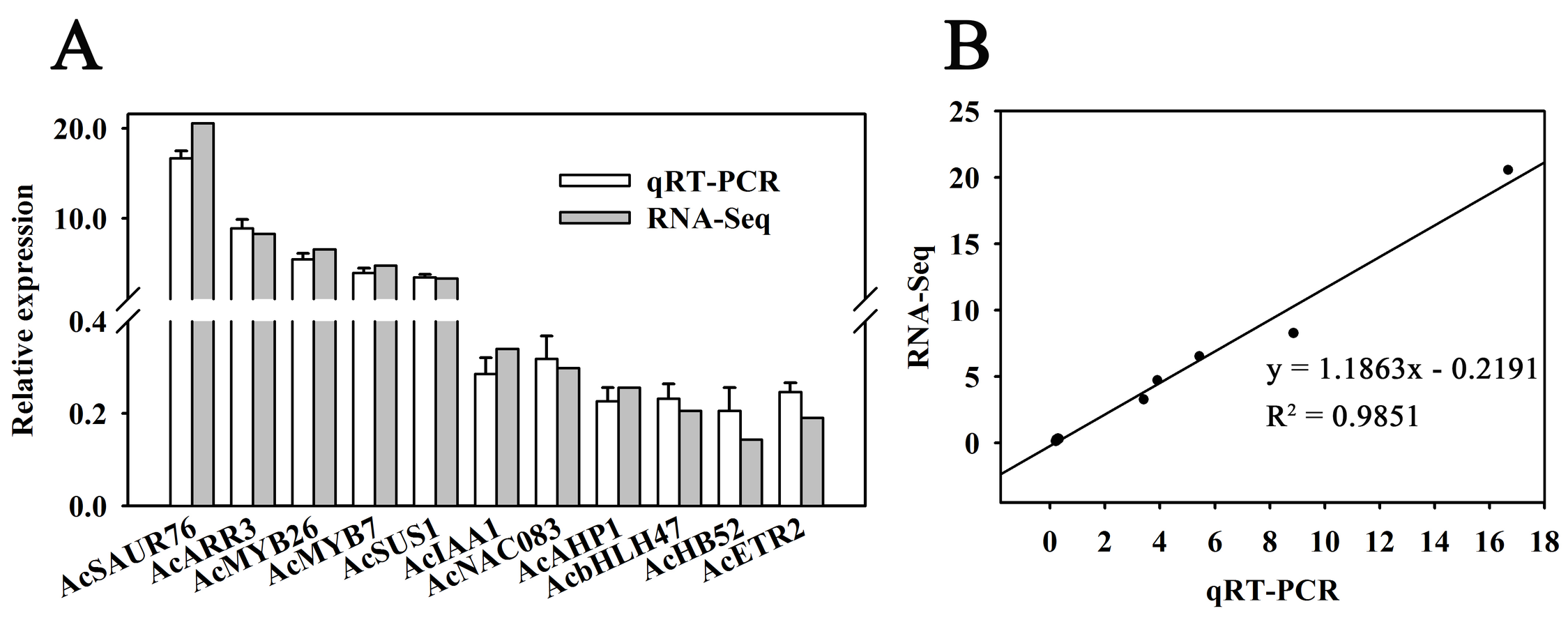

Supplement: S2 Fig — (TIF) [file pone.0240355.s002.tif]
