# Supplementary material for: Transcriptome sequencing and endogenous phytohormone analysis reveal new insights in CPPU controlling fruit development in kiwifruit (Actinidia chinensis)
Source: PLoS One. 2020 Oct 12;15(10):e0240355. doi: 10.1371/journal.pone.0240355 (PMC7549808; doi:10.1371/journal.pone.0240355)
Supplement: S2 Table — (DOCX) [file pone.0240355.s004.docx]

**Table S2. Primer list.**

| Gene ID | Forward primer | Reverse primer |
| --- | --- | --- |
| *AcSAUR76*  *AcARR3*  *AcMYB26*  *AcMYB7*  *AcSUS1*  *AcIAA1*  *AcNAC083*  *AcHP1*  *AcbHLH47*  *AcHB52*  *AcETR2*  *AcGA20OX1*  *AcDELLA*  *AcPP2C*  *AcSAUR50*  *AcMIOX1* | ACGACTCCATCACCGTTGTC  CGGATCGACAGGTGTTTGGA  TTGTCCATATGACCCGCACG  GTGGGGTTGCATAGAGGTCC  CTCGCGTTTTGAAGTCTGGC  AGATGGCCAAGAAGTCACCG  TGGGACTTGCCTGGTGATTC  GCAACTATTGCGACGAGCA  GATGGAATACGGAGGCCGAG  AGGCTAACCCAAGACCAAGTG  GGATCTCCACATGCCGGATT  GTCGAGCGATCACACTACCG  AGAACGACGGCTGTCTGATG  TGATCTTGCTCCAAGCGAGG  CGAGTGACCGAGTTGGTTGA  TGTGTCACCTACAACAGCCC | GCACAGCCCACGTACCTATT  CGGCGACTTCCTCTTGTTGA  GCCACGGCAATAACATCGAC  TCCCTCTCTTGATGTCCGGT  AAGGCGTGAGCAATGGTACA  ATCAACGGTCTCCGAGAAGC  ATCCCCACAACTTGGTTGCT  GCCGTTGGATTCATCGACTT  CTGTCGAACAGCGTTTCTGC  GGACACCGAGTTGTTGAGCA  TTCATCGCCACTTGCCGTTA  GGATGCCTTGCCATTTGTT  GAGTGGCCTTGACTCGTTGA  CCTTCCATGGCGTCCTTCTT  ATTTGAACCCGGGACGACTC  GATCCCGACTTGGATGAGCC |
